# Highly elastic conductive polymeric MEMS

**DOI:** 10.1088/1468-6996/16/1/015003

**Published:** 2015-01-28

**Authors:** J Ruhhammer, M Zens, F Goldschmidtboeing, A Seifert, P Woias

**Affiliations:** 1 Laboratory for Design of Microsystems, Department of Microsystems Engineering—IMTEK, University of Freiburg, Georges-Koehler-Allee 102, 79110 Freiburg, Germany; 2 Gisela and Erwin Sick Chair of Micro-optics, Department of Microsystems Engineering—IMTEK, University of Freiburg, Georges-Koehler-Allee 102, 79110 Freiburg, Germany

**Keywords:** benchtop micromolding, conductive PDMS, polymeric MEMS, capacitive strain gauge, highly elastic, antisticking

## Abstract

Polymeric structures with integrated, functional microelectrical mechanical systems (MEMS) elements are increasingly important in various applications such as biomedical systems or wearable smart devices. These applications require highly flexible and elastic polymers with good conductivity, which can be embedded into a matrix that undergoes large deformations. Conductive polydimethylsiloxane (PDMS) is a suitable candidate but is still challenging to fabricate. Conductivity is achieved by filling a nonconductive PDMS matrix with conductive particles. In this work, we present an approach that uses new mixing techniques to fabricate conductive PDMS with different fillers such as carbon black, silver particles, and multiwalled carbon nanotubes. Additionally, the electrical properties of all three composites are examined under continuous mechanical stress. Furthermore, we present a novel, low-cost, simple three-step molding process that transfers a micro patterned silicon master into a polystyrene (PS) polytetrafluoroethylene (PTFE) replica with improved release features. This PS/PTFE mold is used for subsequent structuring of conductive PDMS with high accuracy. The non sticking characteristics enable the fabrication of delicate structures using a very soft PDMS, which is usually hard to release from conventional molds. Moreover, the process can also be applied to polyurethanes and various other material combinations.

## 1. Introduction

Microstructuring of silicon with structures in the micron and submicron range, high aspect ratios, and an abundance of designs led to the triumphant advance of MEMS technology. Over the last few decades, a technology to mechanically structure various materials was developed. While a tremendous number of processes evolved for rigid materials, only a few processes have been established to structure elastic and highly flexible materials such as polydimethylsiloxane (PDMS). Even fewer processes allow one to combine conductive and nonconductive PDMS in one structure to construct flexible actuators, sensors, and other electronics.

PDMS materials are inherently nonconductive, but by mixing conductive particles with the base polymer, conductivity can be achieved. This material mix acts as a basis for the design of functional MEMS elements embedded into an elastic structure. Various different particles have been used to prepare conductive PDMS composites such as carbon-black-filled PDMS (C-PDMS), multi walled-carbon-nanotube-filled PDMS (MWCNT-PDMS), and PDMS filled with silver flakes or particles (Ag-PDMS). The huge variety of conceivable applications for elastic and electrically conductive PDMS devices with embedded functional MEMS elements ranges from strain gauges [1, 2] and pressure sensors [3] to microfluidic sensors [4]. Yet the fabricating of thin and delicate conductive structures with high aspect ratios is still challenging.

One of the most frequently used methods for micro- and nanostructuring of PDMS is soft lithography [5], where a lithographically patterned photoresist is used as a mold for PDMS [6]. This method has been applied to microcontact printing and replica molding of three-dimensional structures [7]. By combining soft lithography with standard lithographic methods, precise microstructures in the lower micrometer range can be obtained [8]. Moreover, soft lithography can also be used to structure and embed conductive PDMS into nonconductive PDMS, as shown by Niu *et al* [9]. After curing the conductive PDMS, the remaining photoresist is stripped and replaced by a bulk of nonconductive PDMS. Chuang *et al* [10] pursued an approach that filled a predefined and molded PDMS structure fabricated using the previously mentioned soft lithographic methods with a PDMS/Cu mixture. The channels inside the PDMS are filled with the conductive PDMS mixture, and excess material is removed using a razor blade. A similar method was described by Agar *et al* [11], where the conductive PDMS is used as a stretchable, electrically conductive adhesive to mount micron-sized discrete electrical components directly onto PDMS. Gerrat *et al* [12] presented an in-plane method to fabricate high-aspect-ratio conductive and nonconductive PDMS structures. The free-standing, all-polymer MEMS structures are realized by a multistep process that involves two deep reactive-ion etching (DRIE) steps.

All processes described in this section are costly and time-consuming. Complex process steps are necessary, and they often cannot be undertaken in a simple lab environment because they require clean room equipment. In addition, many methods use master molds that are either destroyed during PDMS structuring or can only be reused a limited number of times. In this work, we describe a novel process for structuring conductive and nonconductive PDMS and other highly elastic and flexible materials. The main advantages of this process are its benchtop approach and cost-efficiency by replication of an expensive master mold. Elastic structures with an extremely high aspect ratio can be realized. The simple, three-step approach is well suited for rapid prototyping and small batch production. Moreover, in addition to PDMS machining, the process can be applied to various other elastic materials and material combinations. The process has been successfully tested with various silicones and polyurethanes.

Structures with widths 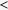
40 *μ*m and heights 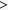
200 *μ*m have been successfully developed using this method. A single structured master (e.g., a DRIE etched silicon wafer) may be used to create multiple casts that can be used as replication molds. Each replication mold can be used to replicate hundreds of reusable PS/PTFE molds to produce the desired structure. Every PS/PTFE mold can be used numerous times, leading to thousands of replications originated from a single wafer.

## 2. Approach

To show the feasibility of the process, we prepared different mixtures of conductive particles added to a very soft PDMS base material. Conductivity is attained by use of carbon black powder, MWCNTs, and silver flakes. However, to achieve conductivity, various other admixtures are conceivable and do not impair the fabrication process itself. The electrical properties of these three different mixtures are measured with a special focus on conductivity under strain. As a possible application for the process, we finally fabricated a highly elastic strain gauge, and we showed the results of the electrical measurements under high strain.

Microstructuring of conductive PDMS is achieved by micromolding, where a negative mold of the desired structure is used to define the geometry of the polymer. This negative mold is frequently made of a thick film resist coated on a silicon or glass substrate. Alternatively, molding micro-structures directly etched into silicon or glass is the method of choice. In both cases, conductive PDMS is poured onto the micromold and excess composite is scraped off using a blade or a similar tool. Nonconductive PDMS is then poured on top of the conductive PDMS as an insulating carrier layer. After cross-linking, the PDMS carrier layer can be peeled off the mold, along with the conductive layer. This method is often referred to as soft lithography. Another way to pattern conductive PDMS is microcontact printing. In this case, a micro fabricated printing mold (e.g., poly(methyl methacrylate)), is used to transfer a thin layer of conductive PDMS from a carrier to a glass slide. In a subsequent step, nonconductive PDMS is poured on top of the imprinted conductive PDMS. After curing, the PDMS layer can again be peeled off, along with the printed microstructures.

Both micromolding approaches, either by using structured resist or etched structures in a substrate, have their downsides. During removal of excess polymer, the master mold is often damaged or scratched, and it is difficult to thoroughly separate small pitched microstructures. In the case of microcontact printing, a printing mold has to be fabricated and only thin layers of conductive PDMS can be transferred, limiting the aspect ratio. Unclear structures at the edges are another disadvantage, because they affect the patterning quality. For both methods, it is a challenge to use very soft PDMS, since it tends to stick to the surface of the master mold or glass slide in the case of microcontact printing. In some cases, the problem can be solved by using appropriate release agents.

For the various reasons discussed above, we developed a three-stage molding technique that transfers a micropattern from a master into an all-elastomer functional structure. To protect the original master from damage during the soft lithography step, the master is first transferred into a soft polymeric negative copy. In the second step, this negative copy is transferred into a hard polymeric positive copy, which can be used for the final soft lithography step. A silicone that can be easily detached from the master is used as the soft polymer. For the hard polymer, a mixture of polystyrene (PS) and polytetrafluoroethylene (PTFE) is applied. This mixture offers improved release properties without any need of a release agent, and it enables a cheap replication process with high reproducibility. Using a soft polymer as an intermediate step is needed, since the hard polymer cannot be released from the master in most cases. For this intermediate step, an easily detachable silicone is preferred. Adding PTFE is the key factor and novelty in this process, allowing one to achieve the essential release features of the hard polymeric copy. The resulting polymeric mold can be fabricated using any preform that can be transferred to silicone. Different materials such as silicon, glass, wax, metal, and various others can be used. In addition, the obtained polymer mold can also be used for patterning other soft polymers, like polyurethanes. Furthermore, the antisticking characteristics of the mold allow the fabrication of thin structures without rupture during peel-off.

## 3. Fabrication

### 3.1. PS/PTFE solution

**Figure 1.**
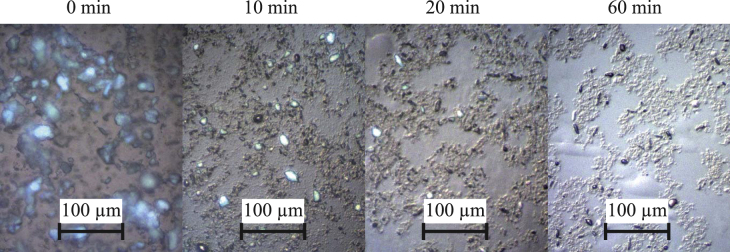
Optical microscope images of PS/PTFE dissolved in GBL and mixed for different time periods. Longer mixing times result in good dispersion of PTFE particles and the formation of bar-like particles.

The PS/PTFE needed for the mold is prepared by first dissolving PS in gamma-Butyrolactone (GBL) in a 25:75 wt% ratio (PS:GBL). GBL was chosen as the solvent for two main reasons: GBL does not swell or attack PDMS, and GBL can be evaporated completely in a fume hood at a relatively moderate temperature [13]. The PS/GBL mixture is kept in a shaker for 48 h to allow a complete dissolution of the PS particles. The resulting solution can be stored for several months at room temperature. The unique easy-release feature of the resulting mold is achieved by adding PTFE to the PS/GBL solution. PTFE powder (Zonyl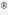 MP 1000, DuPont^TM^, Wilmington, USA) is added in a 5:1 wt% ratio of PS:PTFE, by adding 4.76 wt% to the PS/GBL solution. The antisticking effect of the PTFE increases when a good dispersion of the PTFE powder or a partial unwinding of PTFE crystallites, creating fibrils, is achieved. The creation of fibrils is also referred as fibrillation [14]. To avoid agglomerates and to achieve a good dispersion of the PTFE when stirring the mixture, high shear forces are needed. In this work, a custom-made mixing head [15] was used to stir at 2000 rpm for 30 min, leading to a high degree of dispersion when the PTFE powder is ground up between the mixing head and the walls of the mixing beaker. Figure 1 shows microscopic images of PS/PTFE solutions mixed for different time periods. At longer mixing times, the PTFE particles are ground up and transformed into small, bar-like particles.

### 3.2. Fabrication of a PS/PTFE mold

**Figure 2.**
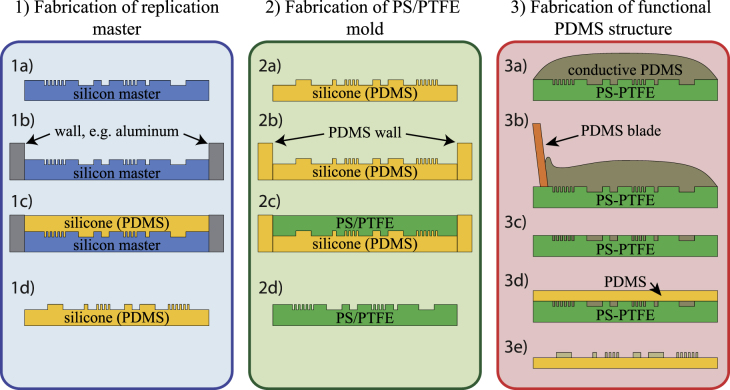
Fabrication procedure consisting of three main steps: 1(a)–(d) Fabrication of a PDMS replica from a Si master substrate. 2(a)–(d) Taking the PDMS replica as a mold to obtain a PS/PTFE mold. 3(a) Pouring conductive PDMS on PS/PTFE master. 3(b)–(c) Removing excess material using a PDMS blade. 3(d) Pouring nonconductive PDMS atop of the uncured conductive PDMS structures. 3(e) Demolding cured condcutive and nonconductive PDMS structure.

The subsequent fabrication process allows the production of PDMS structures with very high aspect ratios. In this work, structures with a 1:10 aspect ratio have successfully been developed, but even higher ratios are feasible. The entire fabrication of PDMS structures with this process is depicted in figure 2.

The basic prerequisite for this PDMS fabrication process is a structured hard substrate with a negative copy of the desired conductive PDMS structure. If a sensor or actuator is to be created, the negative copy should form the conductive structures of this sensor. The substrate may either be a silicon wafer etched by DRIE or a lithographically structured photoresist. In principle, anything that can be molded and detached from PDMS can be used.

In a first step, a negative PDMS copy of the master substrate is generated, as seen in figure 2, 1(a)–(d). To allow an easy and gentle handling of the master substrate, we recommend applying an appropriate release agent to its surface. Silicon wafers, for instance, may be coated with a silane thin film to allow easy detachment. In this work, silicon master wafers were incubated with 50 *μ*l of (tridecafluoro-1,1,2,2-tetrahydrooctyl)silane (ABCR Dr. Braunagel GmbH & Co. KG, Karlsruhe, Germany) for 8 h to deposit a silane thin film.

Once the silane film or a comparable release agent has been applied to the master substrate, a silicone is poured onto the structures to be replicated (figure 2, 1(c)). This silicone should provide easy handling features, good detachment characteristics, sufficient stiffness to prevent deformation, and good temperature stability up to 140 °C. We used Sylgard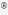 184 (Dow Corning Corp., Midland, MI, USA) mixed in a 10:1 ratio (base:curing agent), which has a shore hardness of A 50. After curing, a negative replica of the structure can easily be removed (figure 2, 1(d)).

In a subsequent step, a PS/PTFE mold is created, as seen in figure 2, 2(a)–(d). The PDMS replica, which was fabricated in step 1, needs to be installed in a PDMS frame to form an open mold for the dissolved PS/PTFE (figure 2, 2(b)), as explained above. Preferably, the same PDMS that has been used for the replica itself should be used to create a frame with a height of a few millimeters.

The PS/GBL/PTFE mixture is poured into the mold (figure 2, 2(c)). To build a PS/PTFE mold that is thick enough for handling, the mold should have a depth of approximately 8 mm and it should be filled up to the upper edge, thus resulting in a 2 mm-thick PS/PTFE replica after hardening (i.e., solvent removal). Hardening of the PS/PTFE mold requires the complete removal of GBL, which is achieved for this 8 mm-high filling through a five-step baking sequence, displayed in table 1. Regardless of the 75% reduction in volume when evaporating GBL, a highly accurate PS/PTFE master mold is obtained and no signs of shrinking are observed in the resulting PS/PTFE structure.

**Table 1.** Baking sequence for PS/PTFE mold hardening.

Step	Description	Temp. in °C	Time
1	Preheating	100	10 min
2	Soft-bake	110	4 h
3	Heat up	140	10 min
4	Hard-bake	140	100 h
5	Cool down	25	1 h

**Figure 3.**
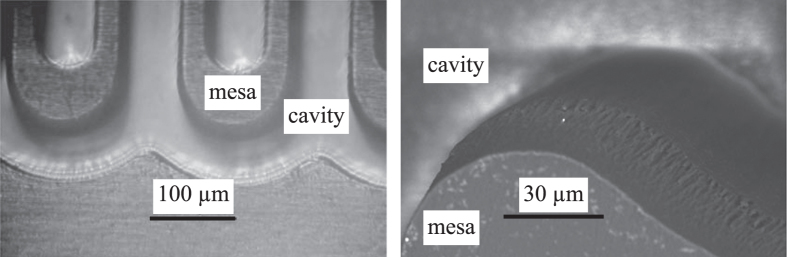
(left) Optical microscope image of a PS/PTFE mold after detachment. The smallest features have a size 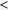
40 *μ*m. (right) SEM image of a PS/PTFE mold.

After completion of baking step 5, the newly created mold can be detached from the PDMS frame by applying light pressure on the back of the frame. The frame with the PDMS replica of the silicon master can be reused for further fabrication of PS/PTFE molds. Figure 3 shows an optical and a scanning electron microscopic (SEM) image of a PS/PTFE mold. In the SEM image, one can see that the upper part of the side wall is slightly rougher than the lower part as a result of the DRIE process.

### 3.3. Preparation of conductive silicones

A variety of conductive PDMS mixtures were prepared to show the flexibility and feasibility of the process and the independence of the material mixes used. The filler has a huge influence on the resulting conductivity, on the maximum allowable strain while maintaining conductivity, and on the stiffness of the compound. Metal fillers in general lead to higher conductivity but are more difficult to disperse due to the huge difference in density between the particles and the base elastomer. MWCNTs show characteristics similar to carbon black with respect of loading and conductivity.

In the present work, MWCNTs, carbon black, and silver particles were used as conductive fillers. For all three mixtures, a very soft, highly stretchable PDMS (Ecoflex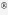 Supersoft 0030, Smooth-On, Inc., Pennsylvania, USA), which can be stretched up to 900% without rupture, was used as base elastomer.

It is important to apply high shear forces during mixing of the fillers to achieve good particle dispersion without large agglomerations. This will yield a smooth and homogenous material.

*C-PDMS.* To prepare the C-PDMS, ENASCO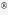 250 P (TIMCAL Ltd., Bodio, Switzerland) was used as filler, as it showed a good conductivity for the lowest loading among all the carbon blacks we tested [15]. To avoid clogging the mixer, the base elastomer was mixed in a 1:1 weight ratio from its two components and mixed with 10.6 wt% carbon black, along with *n*-heptane as a solvent, until the desired viscosity was reached. The mixture was then stirred with a custom-designed mixing head [15] at 2000 rpm for 15 min. To avoid premature curing during mixing, it was cooled in an ice water bath.

*MWCNT-PDMS.* The preparation of MWCNT-PDMS is similar to the preparation of C-PDMS. MWCNTs, Baytubes® C 150 P (Bayer MaterialScience AG, Leverkusen, Germany), were mixed together with the two components of the base elastomer, but this time chloroform was used as a solvent, since a better dispersion of the nanotubes can be achieved, as seen in [16]. The weight ratio of the MWCNTs in the base elastomer was slightly lower than that of carbon black at 7 wt%. The mixing process is conducted the same as it is for carbon black.

*Ag-PDMS.* The preparation of Ag-PDMS, silver powder 99.9 %, 1–3 *μ*m (Fox Chemicals, Pfinztal, Germany), is different from the preparation of the two carbon-based PDMS compounds. After mixing the base polymer, the silver powder is added with 80 wt% and slightly mixed without the addition of any solvent. The crumbly mixture is then ground up manually with a spatula on a glass plate until a smooth compound is obtained.

### 3.4. Fabrication of conductive structures

Once a PS/PTFE mold has been built as described in the section above, it can be used to manufacture multiple conductive PDMS structures. The preferred conductive silicone is prepared and cast into the PS/PTFE mold, as shown in figure 2, 3(a). In the case of MWCNT-PDMS, chloroform must first be evaporated from the mixture in a desiccator before casting the conductive silicone into the PS/PTFE mold, to prevent dissolution of the mold. A soft PDMS blade is used to fill the conductive PDMS into the structures (figure 2, 3(b)).

Before curing the filled structures, a thin layer of pure, nonconductive PDMS is poured on top of the filled mold. During the curing process, the nonconductive PDMS layer cross-links with the conductive structures. This forms a mechanically stable structure that is easily released from the mold.

## 4. Results and discussion

### 4.1. Fabrication process

**Figure 4.**
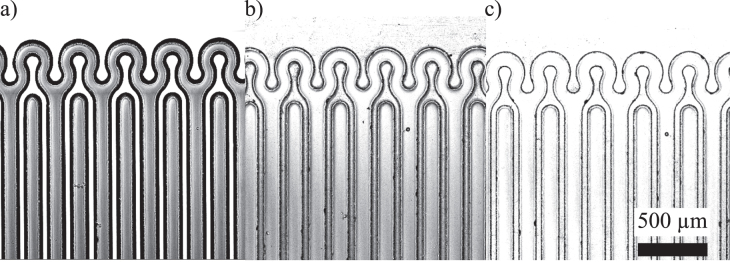
Laser-scanning images of (a) the Si master, (b) the PS/PTFE mold; and (c) the final conductive PDMS structure. The dimensions are as follows: line width, 140 *μ*m; gap width, 40 *μ*m; depth, 220 *μ*m; and inner radius of the meanders, 48 *μ*m.

The process presented in this work allows the fabrication of PDMS structures with very high aspect ratios and minimal structures of 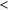
40 *μ*m with a cost-efficient and easily adaptable benchtop approach. For test purposes, a capacitive strain gauge is fabricated. It consists of an interdigital finger structure with conductive lines with a width of 140 *μ*m and gaps between parallel lines of 40 *μ*m. A horseshoe design is used for the electrical connectors on both sides, forming a special stress-tolerant design. The accuracy of the molding process is displayed in figure 4. Using a laser scanning microscope (Axio LSM Pascal 510, Carl Zeiss AG, Oberkochen, Germany), the structural dimensions of the Si master are compared with those of both the PTFE/PS mold and the resulting conductive PDMS structure. The width of the gaps and lines of the structure were measured exemplarily. Within the measurement accuracy of +/− 1 *μ*m, no difference in the dimensions of the Si master mold and the final PDMS structure were observed. Even tiny details, such as scratches or etching defects much smaller than 1 *μ*m, were transferred from the Si master to the PS/PTFE mold.

To characterize the enhanced release features of the PS/PTFE mold, we measured the contact angle in comparison with the corresponding angle of a pure PS mold. For this measurement, we fabricated two planar, circular samples, one with PS and one with PS plus PTFE. The contact angle measurements were performed twice, with the results displayed in table 2. According to Tadmor *et al* [17], the equilibrium contact angle (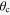) was calculated from the measured advancing and receding angles. The difference between 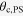 and 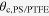 of 36.7° represents a significant increase of the contact angle of the two surfaces [18]. The PS/PTFE blend is much more hydrophobic, which means that PTFE is obviously governing the surface properties with its superior antisticking characteristics, leading to improved release properties.

**Table 2.** Results of the contact angle measurements. The contact angles were measured two times for each sample on different locations. The ‘Results’ column shows the equilibrium contact angle, 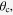 calculated according to [17].

	Advancing contact angle, 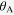		Receding contact angle, 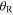		Average		Results
Material	Meas. 1	Meas. 2	Meas. 1	Meas. 2	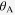	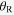	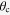
PS	81.2	84.3	27.1	28.5	82.8	27.8	53.3
PS/PTFE	101.3	101.5	78.2	82.8	101.4	80.5	90.0

### 4.2. Material properties

Various different conductive PDMS materials were characterized to evaluate the usability of different sensor and actuator principles. The most important parameter is the electrical resistance during strain. For the characterization, thin film layers of 200 *μ*m of C-PDMS, MWCNT-PDMS, and Ag-PDMS with areas of 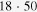 mm^2^ were coated on a layer of nonconductive PDMS (Ecoflex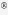 Supersoft 0030). The conductive thin film layers were mounted in a linear stage (LS-110, PI miCos GmbH, Eschbach, Germany) and stretched 2500 times at 5 mm s^−1^, undergoing a maximum strain of 30% for each material. Basically, a maximum strain of 300% could be applied for each material for a few cycles without failure. However, for most applications and requirements, 30% strain is an acceptable value for lifetime tests to prove the performance and high reliability. The resistance was recorded continuously using a four-wire resistance measurement with a digital multimeter (Agilent 34401 A, Agilent Technologies, Santa Clara, CA, USA).

**Figure 5.**
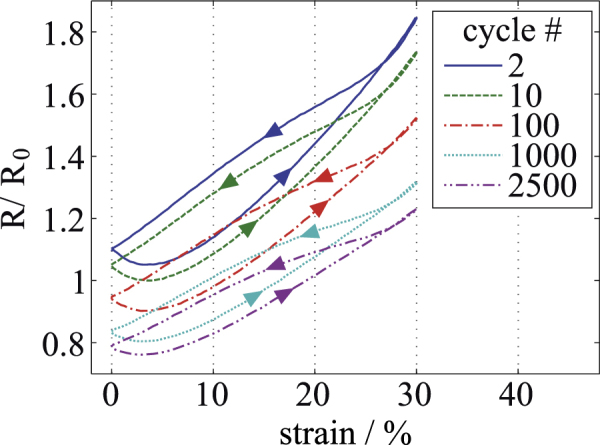
Resistance-strain measurement for a 200-*μ*m thin layer of C-PDMS. Different elongation cycles are shown, starting with cycle 2 after the first preload cycle.

*C-PDMS.* Figure 5 shows the results of the C-PDMS measurements. The base resistance of the strip was measured before stretching to be 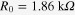 at a distance of 34 mm, leading to a conductivity of 5.1 Sm^−1^. After the first preload cycle, the conductive particles inside the PDMS rearranged, and the resistance increased to 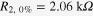 at 0% strain. The measurement results of five representative curves are shown in the figure. The material shows a non linear behavior when stretched and a fairly linear behavior when released, leading to a relatively strong hysteresis. The hysteresis and the absolute values of the resistance decrease slightly with each consecutive cycle. The resistance after 2500 cycles is decreased to 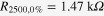 at 0%, strain, thus showing a reduction of 28.6 % after 2500 elongation cycles, in comparison to the resistance after preloading. Possible reasons for the systematic variation of the measurement results during the cycles are a rearrangement of particles during stretching and relaxation, and the viscoelastic behavior of the filled polymer.

**Figure 6.**
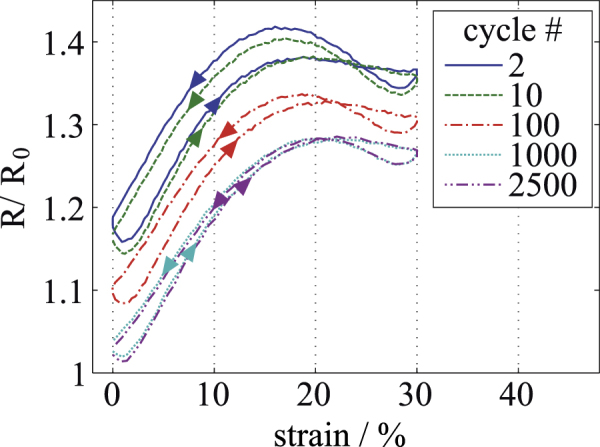
Resistance-strain measurement for a 200 *μ*m thin layer of MWCNT-PDMS. Different elongation cycles are shown, starting with cycle 2 after the first preload cycle.

*MWCNT-PDMS.* Figure 6 displays the measurements taken for MWCNT-PDMS. Starting with a base resistance of 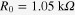, the resistance again increases after the first preload cycle to 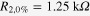 at 0% strain. The base conductivity measured over a distance of 34 mm is 9.0 Sm^−1^. The material shows a similar global behavior to C-PDMS regarding the decrease in resistance and hysteresis with each subsequent cycle. After 2500 cycles, the resistance at 0% strain decreased to 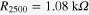, thus yielding to a decrease of 13.6 %, compared to the resistance after preloading. However, MWCNT-PDMS shows a linear behavior only in the low strain region stretching from approximately 2 to 10% strain, which limits its application to resistive strain gauge sensors. A similar curve shape for MWCNT-PDMS regarding the hysteresis was also found by [19].

**Figure 7.**
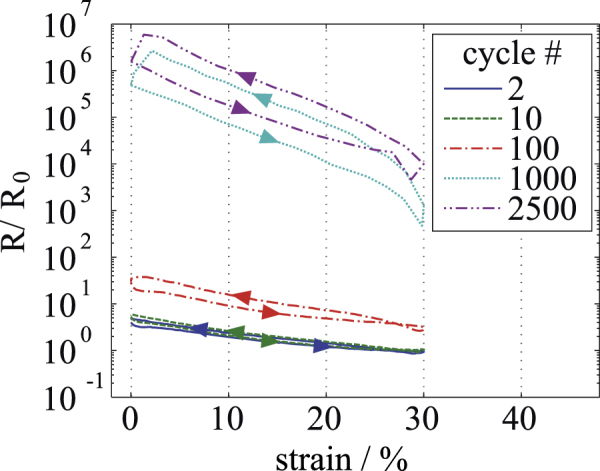
Resistance-strain measurement for a 200 *μ*m thin layer of Ag-PDMS. Different elongation cycles are shown, starting with cycle 2 after the first preload cycle.

*Ag-PDMS.* Ag-PDMS shows a behavior contrary to the ones observed for C-PDMS and MWCNT-PDMS. As displayed in figure 7, the resistance increases with each cycle of lengthening and changes by six orders of magnitude within 2500 cycles, beginning at 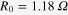 at a distance of 33 mm leading to a conductivity of 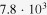 Sm^−1^. The resistance after preloading is 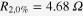. After 2500 elongation cycles, a resistance of 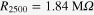 was measured at 0% strain, thus being advanced by a factor of 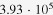 compared to the resistance measured after the preload cycle. Even though the material exhibits the highest conductivity at the beginning, it starts to fail after a few hundred elongation cycles. Its lack of reliability makes it unsuitable for use as sensor or actuator material. Besides that, the resistance of Ag-PDMS decreases with applied strain in a non linear manner. The material shows an increasing hysteresis with each consecutive cycle, leading to a hysteresis that stretches over almost two orders of magnitude for the cycle recorded after 2500 stretch- and-release cycles.

### 4.3. Comparison with the literature

In this section, the conductivity values for different fillers are compared with those found in the literature. We found different definitions for the percolation threshold. Here, we consider the percolation threshold to be the weight percentage of conductive filler material in the polymer matrix directly before a significant increase in conductivity is first observed. The amount of filler is given in weight percentage and has been converted from volume percentage when required (both units are presented). Some of the values have been extracted from graphs. In the articles reviewed, several loading levels were respectively tested. The conductivity values provided in our tables are those with the highest loading levels found in the specific article, since the amounts vary considerably and are otherwise not easily comparable.

Table 3 presents the conductivities for different carbon black fillers. The highest value found for carbon black in the literature we reviewed is 25 Sm^−1^ and was achieved at a loading of 26 wt% [9]. The values depend strongly on the type of carbon black used and the dispersion into the polymer matrix. Table 4 shows the conductivities that can be achieved with MWCNTs. The highest value found for MWCNT-PDMS was 35 Sm^−1^ at a loading of 12 wt% [15]. The values depend strongly on the dispersion method and the aspect ratio of the MWCNTs used. The conductivity usually increases with the aspect ratio [23]. According to [23], the nanotubes could also be over dispersed, leading to a decrease in conductivity. Table 5 shows the maximum conductivities for different types of silver or silver-coated fillers. The highest level found for Ag-PDMS is 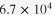 Sm^−1^, which is several orders of magnitude higher than those found for the carbon-based fillers. The percolation threshold depends strongly on the size of the fillers, as shown by [25], where a high level of conductivity can be achieved at a loading of 50 wt%. Furthermore, a strong dependency on shape and dispersion technique can be observed by comparing different sources.

**Table 3.** Comparison of the achieved conductivities and percolation threshold values at zero strain in the literature regarding carbon black.

Carbon black			
Source	Filler specifications	Percolation threshold in wt%	Conductivity in 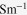
This work	Timcal ENASCO 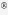 250 P, ∼50 nm [20]	∼5.5	11 wt%: 9.1
Brunne *et al* [15]	Timcal, ENASCO 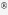 250 P, ∼50 nm [20]	n/a	12 wt%: 9.5
Niu *et al* [9]	Vulcan XC72-R, Cabot Inc., 40–100 nm	∼10	15 wt%: 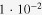 ^a^ 26 wt%: 25^a^
Rwei *et al* [21]	Vulcan XC72-R, Cabot Inc., 40–100 nm	∼1 (0.75 vol%)	6 wt% (4.5 vol%): 0.6^a^
Kontopoulou *et al* [22]	Asbury Carbon, grade 5303, 29 nm	∼0.5	4 wt%: 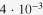 ^a^

a Value extracted from graph.

**Table 4.** Comparison of the achieved conductivities percolation threshold values at zero strain in the literature for MWCNTs.

MWCNTs			
Source	Filler specifications	Percolation threshold in wt%	Conductivity in 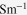
This work	Bayer MaterialScience, Baytubes C 150 P, diam. 13-16 nm, length 1 to 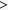 10*μ*m	∼3.5	8 wt%: 9.9
Brunne *et al* [15]	Bayer MaterialScience, Baytubes C 150 P, diam. 13–16 nm, length 1 to 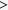 10 *μ*m	n/a	12 wt%: 35
Khosla and Gray [6]	Nanostructure & Amorphous Materials, diam. 60–100 nm, length 5–15 *μ*m	∼1.5	2.5 wt%: 2^a,b^
Khosla and Gray [24]	Cheap Tubes Inc, COOH-functionalized, diam. 10 nm, length 30 *μ*m	∼1.5	4.5 wt%: 23^a^
Liu and Choi [7]	Cheap Tubes Inc., diam. 20–40 nm, no length given	∼6	15 wt%: 6.3

a Value extracted from graph.

b Note: There is a typographical error in the units presented in this work (figure 4). S/m should be S/cm, as one can see by calculating the values from the text and figure 3 and by comparing with [24].

**Table 5.** Comparison of the achieved conductivities percolation threshold values at zero strain in the literature for silver.

Silver			
Source	Filler specifications	Percolation threshold in wt%	Conductivity in 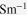
This work	Fox Chemicals, silver powder, APS 1–3 *μ*m, 99.9 %	∼75	82 wt%: 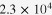
Khosla and Gray [25]	Silver nanoparticles, diam. 80 nm, 99.5 %	∼32.5	50 wt%: 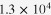
Chung *et al* [26]	NanoAmor, silver nanoparticles, 99 % purity, 90–210 nm	56	65 wt%: 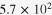
Niu *et al* [9]	Unist Business Corp., silver platelet, 1.2–2.22 *μ*m	∼83	86 wt%: 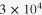 ^a^
Cong and Pan [27]	Silver powder 2 *μ*m	∼ 68 (17 vol%)	73 wt% (21 vol%): 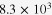
Chuang and Wereley [10]	NanoDynamics Inc, silver coated copper flakes, 10% silver, AC1-0410	∼75	83 wt%: 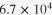 ^a^

a Value extracted from graph.

### 4.4. Functional structure

**Figure 8.**
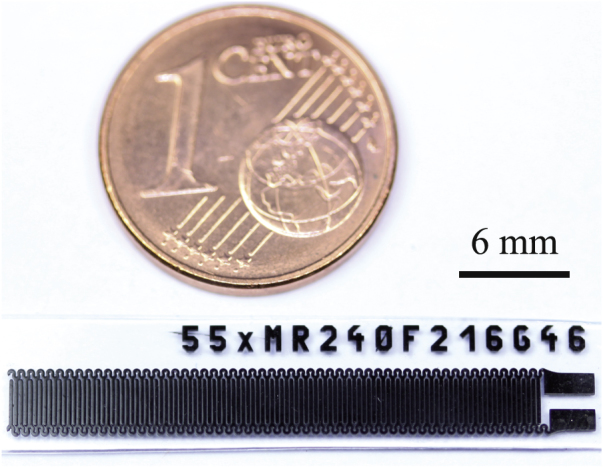
Example of an application of the process with the given materials: a capacitive strain gauge based on an interdigital structure.

**Figure 9.**
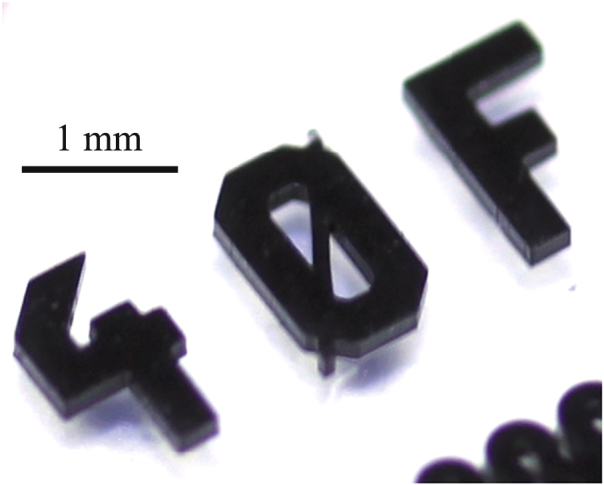
Detailed picture of a C-PDMS structure impressively demonstrating the high aspect ratio achievable with the presented fabrication process.

**Figure 10.**
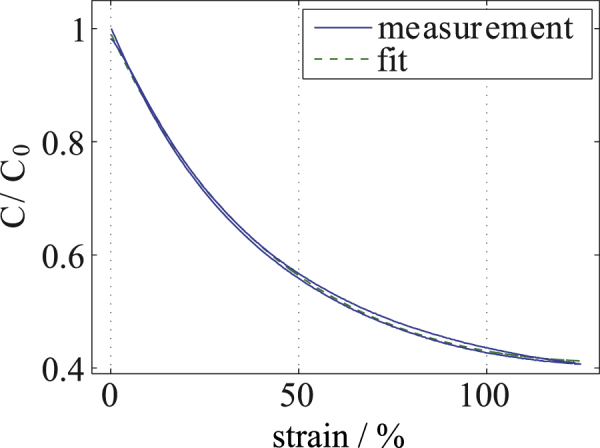
Capacitance-strain measurement for the capacitive strain gauge shown in figure 8 undergoing a strain of 125 %. The fit was calculated using the equation 
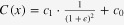
, where *ϵ* is the linear strain.

The conductive properties of the described materials partly limit their use for resistive sensor and actuator principles. However, a suitable application is a capacitive sensor, where the absolute value of the resistance of the conductive material does not play an important role. For example, a capacitive strain gauge has been designed and fabricated with the described process. Figure 8 shows an interdigital structure (IDC) that forms such a capacitive strain gauge. The IDC has 216 *μ*m-wide fingers and 46 *μ*m-wide gaps and was fabricated using C-PDMS. The high degree of accuracy is impressively demonstrated in figure 9, where details such as the type number of the master mold are replicated with excellent precision. The sensor shows a base capacitance of 49.5 pF. An analytical value for the capacitance can be obtained by conformal mapping [28], which leads to a value of 49.1 pF. The sensor can be easily calibrated by absolute strain measurements. For this, the capacitive strain gauge was elongated with a maximum strain of 125 % at a speed of 1 mm S^−1^ as shown in figure 10. At 125 % strain, the capacitance is decreased by 59.2 % down to 20.2 pF. A calibration curve can be obtained by fitting the measured results to the original description of the simple plate capacitor by assuming constant volumes:1
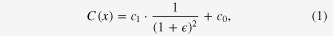
where *ϵ* is the linear strain and *c*_0_ and *c*_1_ are fit parameters.

## 5. Conclusion and outlook

In this work, we presented a novel, cost-efficient, and easy-to-reproduce process to obtain a mold with easy release features. The mold can be used for simple benchtop fabrication of sensors and actuators with filled polymers. Small-batch productions will not impair the master structure. Conductive and nonconductive PDMS structures can be combined, and high aspect ratios of more than 1:10 with minimum feature sizes below 40 *μ*m can be achieved. The process can be also applied to a variety of other materials, such as polyurethanes.

Furthermore, we demonstrated the fabrication process and characteristics of three different conductive PDMS compounds with varying types of conductive fillers. A capacitive strain gauge was developed to demonstrate an appropriate application of the process. The strain gauge was able to measure strains up to 125 %.

This highly flexible process technology can easily be adapted to provide other devices and to employ other materials. Its most attractive characteristics are its low-cost process chain, its material independence, and its high reproducibility in a rapid benchtop approach.

In future work, the process will be used to fabricate highly elastic and stretchable capacitive sensing structures that may be used as strain gauges to measure the mechanical properties of different ligaments. Due to the low elastic modulus of the obtained sensors, the measurements may be carried out with a negligible influence on the anatomical object being studied.
